# Quantitative Analysis of Challenges Encountered by UK Widening Participation Medical Students in Comparison With Their Non-Widening Participation Peers

**DOI:** 10.1177/23821205241249012

**Published:** 2024-05-26

**Authors:** Krishna Chaitanya Ravulapalli, Nicolle M Arroyave Caicedo, Daniel Zahra, Mahrukh Mirza

**Affiliations:** 1 5994 Newcastle University, Newcastle upon Tyne, UK; 2 4919 University College London, London, UK; 3 6633 University of Plymouth, Plymouth, UK

**Keywords:** medical education, inequalities, widening participation

## Abstract

**OBJECTIVES:**

Few studies have captured the experiences of widening participation (WP) medical students, and none have compared their experiences to their non-WP peers. This study aims to identify which challenges WP students are more likely to face.

**METHODS:**

A 22-item questionnaire was distributed to medical students across all UK medical schools. Students were asked yes or no questions on whether they faced challenges in finances, socializing, physical and mental health, academic attainment, and COVID19-related teaching changes.

**RESULTS:**

One-hundred seventy-six medical students from all year groups across the UK responded, with 97 students from a WP background. WP students were significantly more likely to have their personal background impacting their mental health (OR = 2.65, WP  =  0.002), more than twice as likely to feel that their job impacted their studies (OR = 2.53, *P* ≤.05), more likely to feel limited by their financial situation (OR = 2.29, *P*≤.05) and to receive support from student finance (OR = 2.08, *P* < .05).

**CONCLUSION:**

WP students were more likely to face challenges in mental health and finances in medical school compared to their peers. These findings, further informed by qualitative insights can aid in advancing equity in medical training.

## Practice points

1. Widening participation (WP) medical student cohort reported no increased frequency of obtaining a job during medical school compared to their WP peers, but were more likely to feel that having a job would impact their studies.
2. While no statistical significance was reached in rates of adverse mental health between WP and non-WP cohorts, WP students more likely to feel that their personal background adversely impacted their mental health.
3. Despite medical school initiatives, WP medical students were less likely to feel represented by the medical workforce and were more likely to experience discrimination than their non-WP peers.

## Introduction

Widening participation (WP) refers to the process of encouraging underrepresented groups from different socioeconomic and ethnic backgrounds to apply for higher education (HE). Students from these underrepresented groups in medical school are referred to as being from a WP background or as WP students. A recent investigation showed that medical students from families belonging to a lower socio-economic class (LSEC) were the most underrepresented group, with other underrepresented groups including mature, disabled and those from Black, Asian or minority ethnic (BAME) background.^1–3^

Clinical outcomes are worse in patients of minority ethnicities and LSECs due to differences in language, health beliefs, clinical presentations, and clinician bias.^4–8^ To address this issue there are frequent teaching, research, and other clinical initiatives to account for the lack of medical school teaching within these areas.^9–11^ These initiatives are also employed to educate colleagues trained in areas with lower LSEC rates and less ethnic diversity. Despite these efforts, recent systematic reviews concluded that patients of ethnic minority and LSEC backgrounds were still at higher risk of suffering from dosing errors, complications in care, adverse drug effects and healthcare-associated infections.^6,7^ In addition to the above initiatives, promoting racial/ethnic/SEC concordant patient–clinician interaction has emerged as a possible long-term solution to improving patient outcomes in these groups. Increasing diversity in the medical workforce to better match the diversity of the national population can have many benefits on healthcare; patients feeling more connected with their doctors, increased cultural understanding of patients, less feelings of isolation and increased perspectives among the healthcare team.^12^ Considering such research, medical schools and the BMA recognize that cohorts should reflect the society that they will go on to serve.^13–15^ As a result, many medical schools have started WP programs which incorporate contextual admission processes, foundation year programs, and substantial outreach programmes.^16^

However, students from these underrepresented backgrounds are more likely to face extra barriers to HE such as lack of support from parents and schools and are more likely to achieve lower A-level grades.^17,18^ WP students felt less prepared in every aspect of the medical school admissions process when compared with their non-WP peers.^13,19–21^ Many WP students had not been given the chance to achieve their full academic potential and were discouraged from applying to HE.^19^ Under traditional entry requirements, these students are less likely to receive offers from medical schools.

While the WP student experience at the college level (16-18 years old) is becoming increasingly well-documented, there is less research focusing on the experiences of these students in UK medical schools. Research has shown the initial barriers that WP students face when applying to medical school, do not disappear after admission.^18,19^ While the work by Basset et al & Brosnan et al focuses on the experiences of first-in-family medical students, it highlights that financial and social barriers may prevent inclusivity of some WP students in the medical school community.^18,19^ This is supported by findings portraying how WP students find it more difficult to fit in with their peers or identify with staff,^20^ making them less likely to ask for support.^22,23^ In addition to affecting a medical student's sense of belonging in the medical school, these barriers have been shown to affect their studies. Financial worries during medical school,^24^ as well as the discrimination faced by BAME students have the potential to impact exam performance.^25^ BAME students have also shared how they feel pressured to represent their ethnic group in a positive light, a phenomenon known as stereotype threat.^16,23^

Current literature informs us of the challenges UK WP students may face such as feeling out of place, struggling financially, and dealing with discrimination, but fails to compare this against non-WP students quantitatively. We aim to fill these gaps in knowledge by further researching what financial, social, and academic challenges WP students face once they are in medical school. This would allow us to see what areas are in most need of addressing.

## Methods

### Research design

This study uses a cross-sectional survey to collect quantitative data using the JISC online questionnaire platform. This questionnaire was disseminated between 11th June 2021 and 8th January 2022. Sample size was calculated as 66 using the qualitative variable equation in case-control studies described by Charan & Biswas.^26^ There is no data on the prevalence of WP students in medical schools as a whole as opposed to individual characteristics such as socioeconomic status or ethnicity. Therefore data in a previous unpublished study by our group looking into skills on admission to medical school between WP and non-WP students were used to infer prevalence of WP students in the cohort.

### Inclusion and exclusion criteria

Participants were only included if they were a current medical or intercalating medical student at one of the medical schools listed in the demographic questions which included all UK medical schools at the time of launching the survey. All other participants were excluded.

### Data gathering

The questionnaire was disseminated through the social media channels of the Widening Participation Medic Network (WPMN) on Twitter, LinkedIn, Facebook, and Instagram. It was also distributed by local representatives of WPMN on medical school groups. Distribution messaging in all channels was standardized with a message requesting all medical students regardless of WP status to fill out the questionnaire.

### Ethical approval

Ethical approval was sought and approved by Plymouth University—project ID 2712.

### Questionnaire design

The questionnaire assessed the WP status of students using WP criteria by merging the different criteria used by each UK medical school. There are 44 quantitative questions spanning 6 domains: WP criteria, financial barriers, social and community barriers, physical and mental health, academic attainment, and response to COVID19-related teaching changes. Questions were derived based on challenges previously identified by medical students.^19,27,28^ The survey used pre-set answers of Yes or No or a Likert scale. The first page of the questionnaire was a participant information sheet where consent was asked and if participants did not consent then their data will not be included in any analysis and will be deleted. Participants providing consent on the consent page of the questionnaire was considered as informed consent and this method of obtaining informed consent was approved by Plymouth University's Ethics Committee. The last page included a study group email that participants could email to opt-out or ask further questions. The survey used can be seen in Supplemental material.

### Data handling and analysis

Results were split into 2 groups: WP student responses and non-WP student responses, using the criteria set from the answers of section 1, of which one criteria needs to be fulfilled to be defined as a WP participant. Groups were compared to see if one group faced a challenge more than the other. “Yes and no” data was compared between groups using proportions, odds ratio, and Fischer’s exact test. Likert scored questions were analysed using unpaired t-test.

## Results

### Participant characteristics

One-hundred seventy-six UK medical students participated in this study. There were 97 WP and 79 non-WP students across the UK (Table 1). Sixty-five percent of respondents were in the clinical component of their degrees. Thirty-four percent of the respondents were from a minority ethnic background and 60% were female.

**Table 1.** Participant characteristics of medical student cohort.

		WP	Non-WP
Part of medical degree	Preclinical students (years 1-2)	35	25
	Clinical year students (3rd-5th year including intercalation)	62	54
Ethnicity	White	36	79
	Asian or Asian British	36	0
	Black, African, Caribbean or black British	11	0
	Mixed or multiple ethnic groups	10	0
	Other ethnic groups	4	0
Region of medical school	South west	12	5
	South east	15	8
	London	13	9
	Midlands	14	5
	North	10	13
	Wales	14	12
	Scotland	18	27
	Ireland	1	0
Gender	Male	31	18
	Female	64	61
	Non-binary	2	0

### Financial challenges

**Figure 1.**
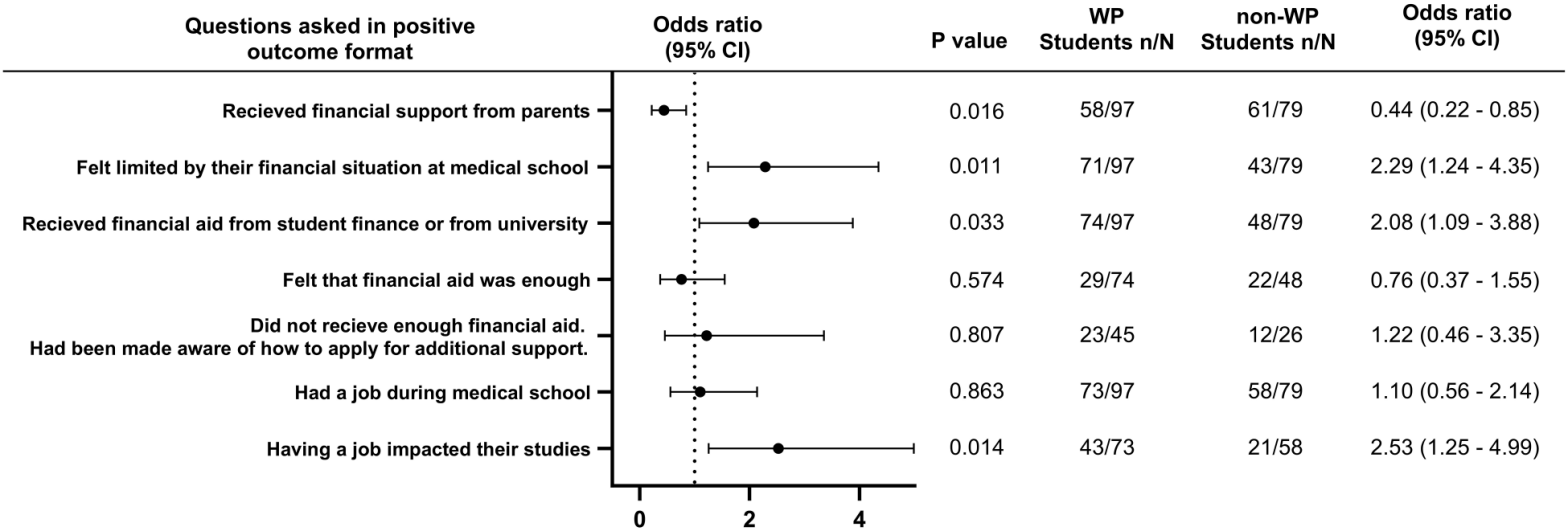
Odds ratio forest plots for financial barriers faced by WP students compared to non-WP students.

Participants were asked 8 questions regarding financial barriers (Figure 1). WP students were over twice as likely to feel limited by their financial situation (OR = 2.29, *P*≤.05), receive financial aid from university or student finance (OR = 2.08, *P* < .05) rather than parents (OR = 0.44, *P*≤.05) and have a job impact their studies (OR = 2.53, *P* ≤.05). There was no significant difference between both groups in receiving enough financial aid (OR = 0.76, *P*  =  .57), being aware of how to receive more financial support (OR = 1.22, *P*  =  .807) and having a job in medical school (OR = 1.10, *P*  =  .86). Forty-six percent of WP students did not receive enough financial aid compared to 32% of non-WP students. Seventy-five percent of WP students had a job during medical school compared to 73% of non-WP students.

### Challenges to feeling part of the medical school community

**Figure 2.**
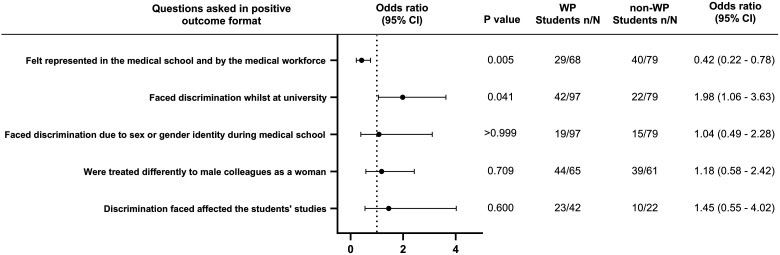
Odds ratio forest plots for medical school community challenges faced by WP students compared to non-WP students.

Participants were asked 6 questions about challenges they may have faced in feeling part of the medical school community (Figure 2 and Table 2). There was no significant difference in feeling part of the medical school community between WP and non-WP students (mean  =  2.25 vs 2.47, *P*  =  .07). WP students were nearly twice as likely to face discrimination at university (OR = 1.98, *P*  =  .041) and to not feel represented in the medical school and by the medical workforce (OR = 0.42, *P*  =  .005). There was no significant difference between groups in facing discrimination due to sex or gender identity (OR = 1.04, *P*≥.999), being treated differently to male colleagues (OR = 1.18, WP  =  .709) and discrimination affecting a student's studies (OR = 1.45, *P*  =  .60).

**Table 2.** Likert scale scoring between WP and non-WP students on how much they feel a part of the medical school community.

	Not at all part of the community—1 (%)	Slightly part of the community—2 (%)	Part of the community—3 (%)	Very much part of the community—4 (%)	Mean (95% CI)	*P*-value
WP students	14%	53%	27%	6%	2.25 (1.09-2.40)	.07
Non-WP students	11%	42%	36%	11%	2.47 (2.28-2.66)	

### Physical and mental health challenges

**Figure 3.**
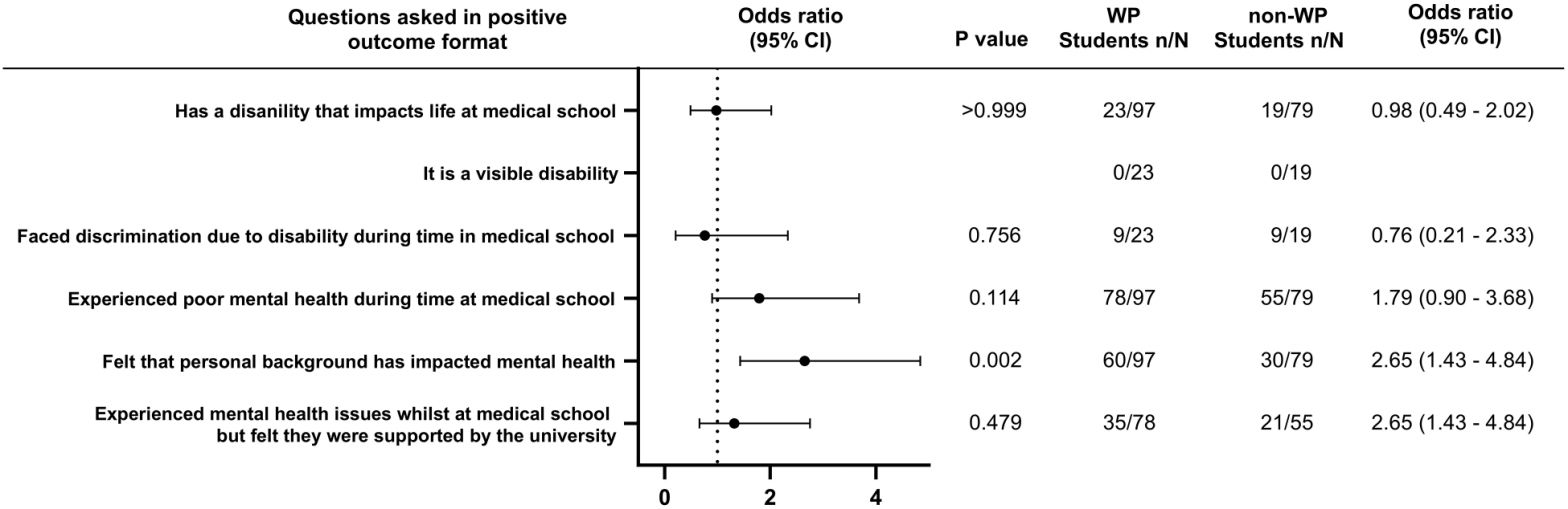
Odds ratio forest plots for physical and mental health challenges faced by WP students compared to non-WP students.

Participants were asked 6 questions about challenges they may have faced due to physical and mental health (Figure 3). WP students were more than twice as likely to have their personal background impacting their mental health (OR = 2.65, WP  =  0.002). There was no significant differences in students having a disability that impacts life (OR = 0.98, WP≥0.99), facing discrimination due to disability (OR = 0.76, *P*  =  .76), experiencing poor mental health (OR = 1.79, *P*  =  .11), and feeling supported by the university if they had poor mental health (OR = 1.32, *P*  =  .48). There were no students in this cohort who had a visible disability.

### Challenges in academic attainment and impact of COVID19-related teaching changes

**Figure 4.**
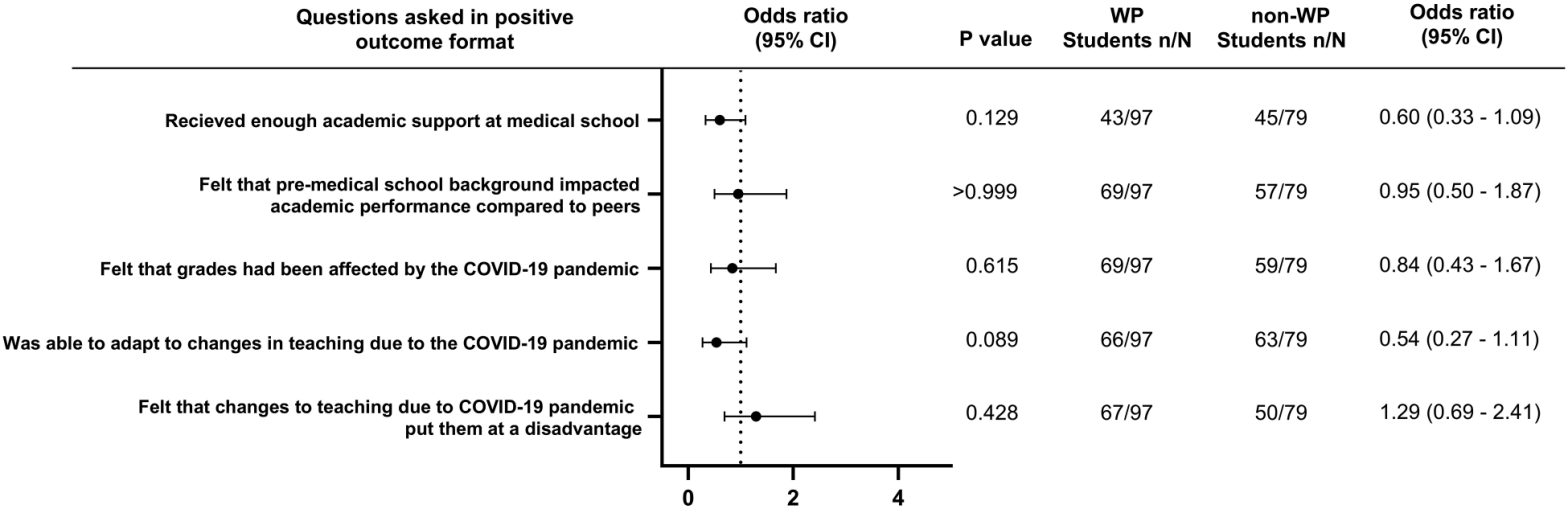
Odds ratio forest plots for academic self-perception and COVID19-related teaching challenges by WP students compared to non-WP students.

Participants were asked 6 questions about challenges they may have faced in academia and the impact of COVID19-related teaching changes (Figure 4 and Table 3). There was no significant difference in students receiving enough academic support (OR = 0.60, *P*  =  .129), feeling that their pre-medical school background has impacted their performance (OR = 0.95, *P*≥.99), feeling their grades were affected by COVID19 (OR = 0.84, *P*  =  .62), being able to adapt to COVID19-related teaching changes (OR 0.54, *P*  =  .089), feeling as though COVID19-related teaching changes put them at a disadvantage (OR = 1.29, *P*  =  .43) and confidence in launching an academic project (mean 4.12 (WP) versus 4.2 (non-WP), *P*  =  .90).

**Table 3.** Likert scale scoring between WP and non-WP students on how confident they feel in launching an academic project (1  =  not confident at all, 10  =  completely confident).

	Mean (95% CI)	*P*-value
WP students	4.12 (3.64-4.67)	.90
Non-WP students	4.20 (3.64-4.77)	

## Discussion

### Financial barriers and work impact

This study presents valuable insight into the WP student medical school experience compared to their non-WP peers. The domain with the biggest difference in experience were barriers in students’ financial situation. WP students were over twice as likely to feel limited by their financial situation, require financial aid, and have their job impact their studies. Only one study has interviewed students of WP background to understand their experiences with financial hardship in the UK^27^ Our study supports the findings by Basset et al, 2019 and identifies these financial hardships are experienced more commonly by WP students nationally.^27^ The cohort recruited in Basset et al, 2019 include some non-WP participants, therefore we cannot ascertain if these experiences are unique to the WP cohort from that study alone. Our study also identified that there was no significant difference between both groups in obtaining a job, however WP students were over twice as likely to feel that having a job would impact their studies. According to Basset et al 2019, students have previously stated that their primary motivation for taking up a job is to provide financial support for their family and to handle the financial instability that accompanies clinical years.^27^ These reasons may indicate that WP students may be undertaking more work than their non-WP colleagues to undertake larger/more important financial goals. This may contribute towards the poor mental health and academic attainment in WP students.^29–31^ The nature of extra-curricular job commitments of medical students and particularly those of WP background should be investigated further to understand if additional support needs to be provided to WP students. Increased financial support through bursary or increased loan may be a way to tackle this.

### Representation and diversity

A considerable percentage of students in both groups (70% from WP backgrounds vs 49% from non-WP backgrounds) do not feel represented by the medical school or the workforce. However, the proportion of WP students who feel unrepresented is twice as high as their non-WP counterparts. This is in line with the views of students at the medical school level where it is felt there is not enough ethnic representation in the senior faculty and in the medical school curricula.^32–34^ This is a recognized issue and there are initiatives to tackle this which aim to create a more ethnically inclusive medical school society.^35^ The possible cause behind the higher likelihood of WP students feeling underrepresented, in contrast to their non-WP counterparts, could be due to ethnic underrepresentation. Notably, a significant proportion of students in both groups who felt unrepresented were white British (42% from WP backgrounds and 100% from non-WP backgrounds). Among these white British students, only one was male and heterosexual, while the remaining students identified themselves as female and/or gay, lesbian, or bisexual. Lack of recruitment of women to senior medical school roles has long been identified as an issue. A recent quantitative study identified that within London medical schools there was no significant difference between the proportion of women in the student body and in the leadership positions.^34,36,37^ In our cohort, no heterosexual female student who felt unrepresented was from a London university. This may suggest that universities outside of London do not have a similar presence of women in the faculty, there may be other reasons this student body do not feel represented or that it is in the medical workforce they feel underrepresented in. The NHS medical workforce is represented in proportion or overrepresented to the national population by South Asian, black and British white ethnicities and underrepresented by east Asian ethnicities. Only 47% (16 of 34 students from south Asian ethnicity) and 9% (1 of 11 students from black, African, Caribbean or black British ethnicity) felt represented from our study. This could suggest that these students felt they were not represented at the medical school level or that the student had not seen representation due to their geographical location.

### Discrimination and racial harassment

Our study reported WP students were nearly twice as likely to face discrimination than non-WP students, however there was no difference in the likelihood of being discriminated against due to sex or gender identity. This is likely due to race as there was no difference in WP students facing discrimination due to sex or gender identity compared to their non-WP colleagues. This further reinforces the findings of recent reports by BMJ and BMA that highlighted the issue of racial harassment, discrimination, and bullying experienced by many ethnic minority students, with insufficient actions taken by medical schools to address it.^38^ A recent project by Lynn et al suggests that student led initiatives to raise awareness of discrimination such as racism and inspiring confidence to advocate against it may be a more effective method than traditional faculty/school led approaches.^39^

### Social integration and mental health

WP students in our cohort on average felt only slightly less part of the medical school community compared to the non-WP cohort, with no significant difference. Although evidence of WP students finding difficulty in establishing social networks exists throughout the literature, another common theme that emerges is that WP students find strong social connections with individuals from similar backgrounds—as identified by Krstic et al.^22^ With medical schools placing more significance on WP programs and receiving increased applications, it is hoped that the situation will improve for WP students in this regard.

Although no significant difference between having or facing discrimination due to mental or physical health and disability was seen between groups, WP students were 2 times more likely to have mental health impacted by personal background. Adverse mental health among medical students is unfortunately common with over 1 in 5 students receiving a formal diagnosis last year and many more suffering from bouts of emotional stress.^40^ The cause of the emotional stress can be complex, compounded by several factors.^41^ Much of the current literature attributes much of the adverse mental health seen in medical students to the medical course, however it may be the case that the etiology of adverse mental health may be different in WP students due to different socioeconomic and cultural backgrounds. A review recently looked into the mental well-being of black university students in the UK and highlighted themes of institutional racism and culture shock as contributing factors towards adverse mental health in these students.^42^ This review did not use any studies with students in medical school, and there is no such research for students of other non-white ethnicities or of those medical students coming from a low socioeconomic background. Further investigation through focused interview study is needed into the mental health of the different WP medical student groups to improve pastoral care programs offered by institutions.

### Academic support and COVID-19 adaptation

While no statistical significance was reached, our results suggest that there still may be a link between WP students and a sense of not receiving enough academic support at medical school. According to Curtis & Smith,^31^ students on a gateway course, which mostly comprised students from a lower socioeconomic background (excluding those from Kings College London, where the proportion of students from a lower socioeconomic background is unclear), performed comparably on medical school exit exams to students on traditional courses when their UCAT and A-Level scores were taken into account. Many of these students were also from a BAME background. However, the comparability in performance was only observed after controlling for A-Level and UCAT scores on admission. It would be interesting to examine whether this pattern holds for WP students on traditional courses, as prior data indicates that certain WP groups, such as ethnic minority students, have shown lower performance in undergraduate and postgraduate exams across various exam formats.^43^ Intake of students in gateway medical courses is increasing however many WP students are admitted in traditional courses where there may not be as many individuals from similar backgrounds to form friendships and learn with. Our results and previous evidence suggest this topic is still not well understood. While the findings of this study did not reach statistical significance, there were suggestive trends indicating that WP students may have faced relatively greater challenges in adapting to the changes brought about by COVID-19 in teaching methods. The *P*-value of .09 points towards this potential trend. A study of 2721 medical students found that barriers to online teaching included poor internet connections, distractions from family, and lack of space, which may be particularly challenging for students from low SES backgrounds with family responsibilities.^44^ Understandably, both student group felt their grades were impacted by the pandemic however further investigation through focused interview studies can reveal if WP students may need further support to adapt to online teaching.

## Limitations

There are several limitations to this study approach. While efforts were made to disseminate the survey widely, this studies sample size of 176 students may not fully represent the diverse range of experiences among medical students in the United Kingdom. The data is self-reported without elaboration of why the answers, which may introduce recall bias or participants providing responses they deemed socially acceptable. There is also the possibility of response bias which may impact the representativeness of the sample and the generalizability of the findings to the wider medical student population. Additionally, the survey used in this study is not validated as no previous validated survey exists for this use and it was not pilot-tested.

## Conclusion

Our findings suggest that WP students have a distinct experience during their time in medical school in comparison to their non-WP peers. In order to gain a more comprehensive understanding of the issues faced by WP students, further investigation through qualitative studies is required. Specifically, it is important to explore areas such as financial experiences, discrimination, mental health, and academic support received by WP students.

Collaboration between educational institutions, policymakers, and medical professionals stands as a pivotal pathway for implementing effective changes. These changes, informed by qualitative insights, can pave the way for a medical education system that ensures equal opportunities for every student, irrespective of their background. As we advance towards a more equitable future, understanding the experiences of WP students is not only essential for their success but also integral to advancing the quality of medical education as a whole.

## Supplemental Material

sj-docx-1-mde-10.1177_23821205241249012 - Supplemental material for Quantitative Analysis of Challenges Encountered by UK Widening Participation Medical Students in Comparison With Their Non-Widening Participation Peers Supplemental material, sj-docx-1-mde-10.1177_23821205241249012 for Quantitative Analysis of Challenges Encountered by UK Widening Participation Medical Students in Comparison With Their Non-Widening Participation Peers by Krishna Chaitanya Ravulapalli, Nicolle M Arroyave Caicedo, Daniel Zahra and Mahrukh Mirza in Journal of Medical Education and Curricular Development

## References

[1] Garrud P . Medical schools council: Selecting for excellence - executive summary. 2014. Accessed December 22, 2023. https://www.medschools.ac.uk/media/1203/selecting-for-excellence-final-report.pdf

[2] Medical Schools Council. Selection alliance 2018 report. 2018; (November). Accessed December 22, 2023. https://www.medschools.ac.uk/media/2536/selection-alliance-2018-report.pdf

[3] Medical Schools Alliance. Selection alliance 2019 report. Accessed December 22, 2023. https://www.medschools.ac.uk/media/2608/selection-alliance-2019-report.pdf

[4] Alhomoud F Smith F Soraya Dhillon P Aslanpour Z . Medication use and medicine-related problems (MRPs) experienced by South Asian (SA) and Middle Eastern (ME) patients with chronic diseases in primary care in the UK under the supervision of professor. 2014.

[5] Alhomoud F Dhillon S Aslanpour Z Smith F . South Asian and Middle Eastern patients’ perspectives on medicine-related problems in the United Kingdom. Int J Clin Pharm. 2015;37(4):607-615. doi:10.1007/s11096-015-0103-6 25822040

[6] Chauhan A Walton M Manias E , et al. The safety of health care for ethnic minority patients: A systematic review. Int J Equity Health. 2020;19(1):3-21. doi:10.1186/s12939-020-01223-2 PMC7346414 32641040

[7] Cheraghi-Sohi S Panagioti M Daker-White G , et al. Patient safety in marginalised groups: A narrative scoping review. Int J Equity Health. 2020;19(1):4-16. doi:10.1186/s12939-019-1103-2 PMC7014732 32050976

[8] Roberts BW Puri NK Trzeciak CJ Mazzarelli AJ Trzeciak S . Socioeconomic, racial and ethnic differences in patient experience of clinician empathy: Results of a systematic review and metaanalysis. PLoS ONE. 2021;16(3 March):5-9. doi:10.1371/journal.pone.0247259 PMC7928470 33657153

[9] Lynch C . Response to: racism: the other pandemic. Br Med J. Published online June 11, 2020:m2303. doi:10.1136/bmj.m2303

[10] Louie P Wilkes R . Representations of race and skin tone in medical textbook imagery. Soc Sci Med. 2018;202:38-42. doi:10.1016/j.socscimed.2018.02.023 29501717

[11] Nieblas-Bedolla E Christophers B Nkinsi NT Schumann PD Stein E . Changing how race is portrayed in medical education: recommendations from medical students. Acad Med. 2020;95(12):1802-1806. doi:10.1097/ACM.0000000000003496 32379145

[12] Takeshita J Wang S Loren AW , et al. Association of racial/ethnic and gender concordance between patients and physicians with patient experience ratings. JAMA Netw Open. 2020;3(11):e2024583-e2024583. doi:10.1001/jamanetworkopen.2020.24583 PMC7653497 33165609

[13] British Medical Association. The demography of medical schools: a discussion paper. 2004. https://puntsdevista.comb.cat/edicio9/Documents/2004%20UK%20Demography%20Schools%20Medicine.pdf

[14] Heller A . Diversity in the medical workforce: are we making progress? The King’s Fund. Published February 3, 2020. Accessed December 22, 2023. https://www.kingsfund.org.uk/blog/2020/02/diversity-medical-workforce-progress

[15] Patterson R Price J . Widening participation in medicine: what, why and how? MedEdPublish. 2017;6(4):1-15. doi:10.15694/mep.2017.000184 38406455 PMC10885306

[16] Bullock JL Lockspeiser T Del Pino-Jones A Richards R Teherani A Hauer KE . They don’t see a lot of people my color: a mixed methods study of racial/ethnic stereotype threat among medical students on core clerkships. Acad Med J Assoc Am Med Coll. 2020;95(11S Association of American Medical Colleges Learn Serve Lead: Proceedings of the 59th Annual Research in Medical Education Presentations):S58-S66. doi:10.1097/ACM.0000000000003628 32769459

[17] Ball R Alexander K Cleland J . “The biggest barrier was my own self”: the role of social comparison in non-traditional students’ journey to medicine. Perspect Med Educ. 2020;9(3):147-156. doi:10.1007/s40037-020-00580-6 32323114 PMC7283443

[18] Bassett AM Brosnan C Southgate E Lempp H . Transitional journeys into, and through medical education for first-in-family (FiF) students: a qualitative interview study. BMC Med Educ. 2018;18(1):1-12. doi:10.1186/s12909-018-1217-z 29743061 PMC5944111

[19] Brosnan C Southgate E Outram S , et al. Experiences of medical students who are first in family to attend university. Med Educ. 2016;50(8):842-851. doi:10.1111/medu.12995 27402044

[20] Brown G Garlick P . Changing geographies of access to medical education in London. Health Place. 2007;13(2):520-531. doi:10.1016/j.healthplace.2006.07.001 16962817

[21] Sartania N Alldridge L Ray C . Barriers to access, transition and progression of widening participation students in UK Medical Schools: The students’ perspective. MedEdPublish. 2021;10(1):4-11. doi:10.15694/mep.2021.000132.1 PMC10939539 38486567

[22] Krstić C Krstić L Tulloch A Agius S Warren A Doody GA . The experience of widening participation students in undergraduate medical education in the UK: a qualitative systematic review. Med Teach. 2021;43(9):1044-1053. doi:10.1080/0142159X.2021.1908976 33861176

[23] Morrison N Machado M Blackburn C . Student perspectives on barriers to performance for black and minority ethnic graduate-entry medical students: a qualitative study in a West Midlands medical school. BMJ Open. 2019;9(11):e032493. doi:10.1136/bmjopen-2019-032493 PMC6924783 31784444

[24] Ross S Cleland J Macleod MJ . Stress, debt and undergraduate medical student performance. Med Educ. 2006;40(6):584-589. doi:10.1111/j.1365-2929.2006.02448.x 16700775

[25] Orom H Semalulu T Underwood W . The social and learning environments experienced by underrepresented minority medical students: a narrative review. Acad Med. 2013;88(11):1765-1777. doi:10.1097/ACM.0b013e3182a7a3af 24072111

[26] Charan J Biswas T . How to calculate sample size for different study designs in medical research? Indian J Psychol Med. 2013;35(2):121-126. doi:10.4103/0253-7176.116232 24049221 PMC3775042

[27] Bassett AM Brosnan C Southgate E Lempp H . The experiences of medical students from first-in-family (FiF) university backgrounds: a Bourdieusian perspective from one English medical school. Res Post-Compuls Educ. 2019;24(4):331-355. doi:10.1080/13596748.2018.1526909

[28] Southgate E Brosnan C Lempp H , et al. Travels in extreme social mobility: how first-in-family students find their way into and through medical education. Crit Stud Educ. 2017;58(2):242-260. doi:10.1080/17508487.2016.1263223

[29] Billingsley M . More than 80% of medical students with mental health issues feel under-supported, says *Student BMJ* survey. Br Med J. 2015;351:h4521. doi:10.1136/sbmj.h4521

[30] Olaniyan FV . Paying the widening participation penalty: racial and ethnic minority students and mental health in British universities. Anal Soc Issues Public Policy. 2021;21(1):761-783. doi:10.1111/asap.12242

[31] Curtis S Smith D . A comparison of undergraduate outcomes for students from gateway courses and standard entry medicine courses. BMC Med Educ. 2020;20(1):1-14. doi:10.1186/s12909-019-1918-y PMC6942303 31900151

[32] Forrest D George S Stewart V , et al. Cultural diversity and inclusion in UK medical schools. Clin Teach. 2022;19(3):213-220. doi:10.1111/tct.13472 35243769 PMC9313838

[33] Mills S . A reflection on representation in medical school by Samantha Mills, BM6 student. Medically speaking. Published December 2, 2021. https://generic.wordpress.soton.ac.uk/medicallyspeaking/2021/12/02/a-reflection-on-representation-in-medical-school-by-samantha-mills-bm6-student/

[34] Hoque S Baker EH Milner A . A quantitative study of race and gender representation within London medical school leadership. Int J Med Educ. 2021;12:94-100. doi:10.5116/ijme.609d.4db0 34050640 PMC8411335

[35] Haley K Hutch A . Increasing representation in the medical curriculum through student-staff partnership. Irel J High Educ. 2022;14(1):728-729.

[36] Carnes M Morrissey C Geller SE . Women’s health and women’s leadership in academic medicine: hitting the same glass ceiling? J Womens Health. 2008;17(9):1453-1462. doi:10.1089/jwh.2007.0688 PMC2586600 18954235

[37] Kvarner KJ Aasland OG Botten GS . Female medical leadership: cross sectional study. Br Med J. 1999;318(7176):91-94. doi:10.1136/bmj.318.7176.91 9880281 PMC27681

[38] British Medical Association. BMA racial harassment charter for medical schools – review. 2022. https://www.bma.org.uk/media/6264/bma-racial-harassment-charter-review-oct-2022.pdf

[39] Lynn TM D’urzo KA Vaughan-Ogunlusi O , et al. The impact of a student-led anti-racism programme on medical students’ perceptions and awareness of racial bias in medicine and confidence to advocate against racism. Med Educ Online. 2023;28(1):2176802. doi:10.1080/10872981.2023.2176802 36787247 PMC9930825

[40] British Medical Association. Caring for the mental health of the medical workforce. 2019. https://www.bma.org.uk/media/ckshvkzc/20190196-bma-mental-health-survey-report-revised.pdf

[41] Moir F Yielder J Sanson J Chen Y . Depression in medical students: current insights. Adv Med Educ Pract. 2018;9:323-333. doi:10.2147/AMEP.S137384 29765261 PMC5944463

[42] Stoll N Yalipende Y Byrom NC Hatch SL Lempp H . Mental health and mental well-being of black students at UK universities: a review and thematic synthesis. BMJ Open. 2022;12(2):e050720. doi:10.1136/bmjopen-2021-050720 PMC8886426 35228276

[43] Woolf K Potts HWW McManus IC . Ethnicity and academic performance in UK trained doctors and medical students: systematic review and meta-analysis. Br Med J. 2011;342(mar08 1):d901-d901. doi:10.1136/bmj.d901 PMC3050989 21385802

[44] Dost S Hossain A Shehab M Abdelwahed A Al-Nusair L . Perceptions of medical students towards online teaching during the COVID-19 pandemic: a national cross-sectional survey of 2721 UK medical students. BMJ Open. 2020;10(11):e042378. doi:10.1136/bmjopen-2020-042378 PMC7646323 33154063

