# Supplementary figures and images for: Quantitative Analysis of Challenges Encountered by UK Widening Participation Medical Students in Comparison With Their Non-Widening Participation Peers
Source: J Med Educ Curric Dev. 2024 May 26;11:23821205241249012. doi: 10.1177/23821205241249012 (PMC11131392; doi:10.1177/23821205241249012)

Sample Questionnaire


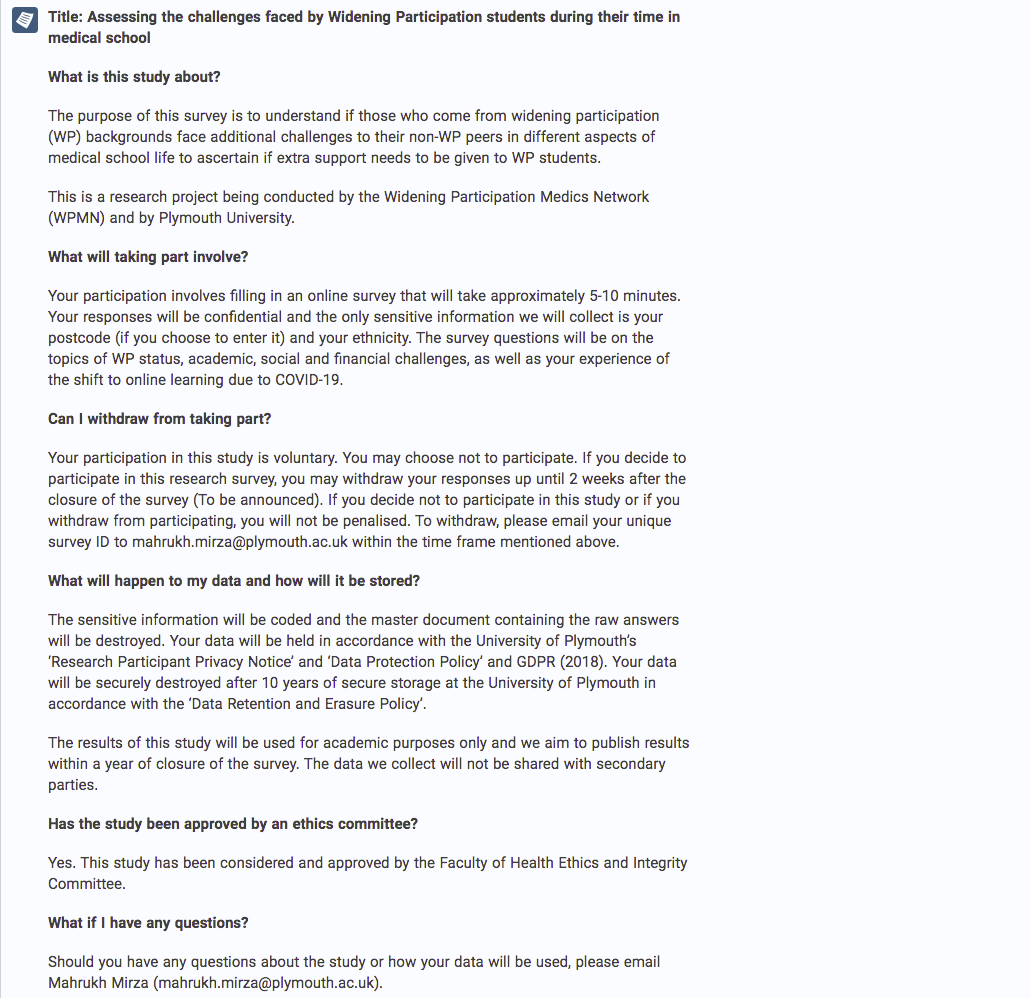


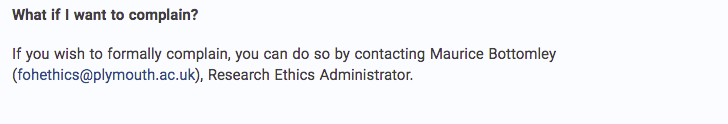


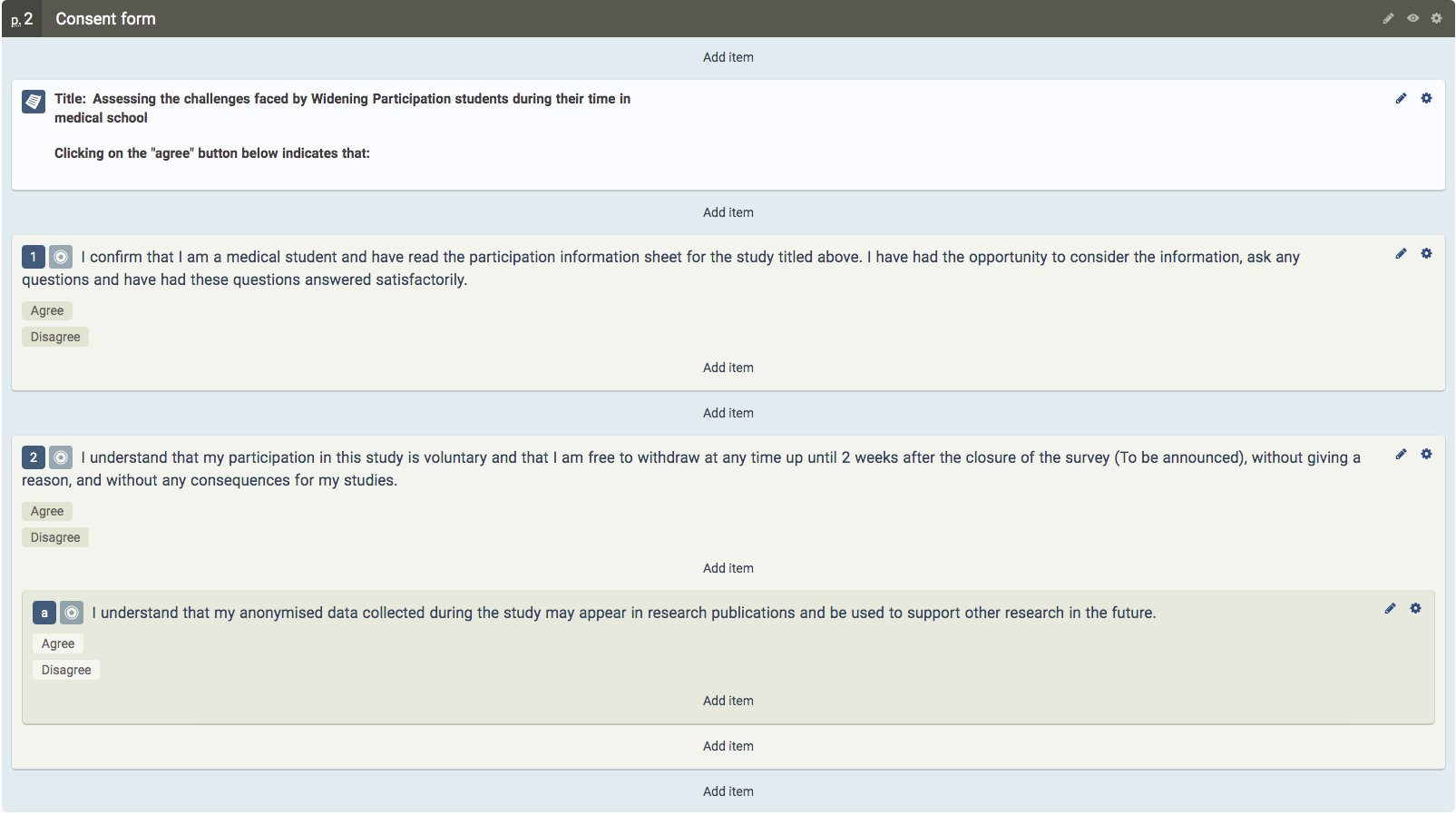


**
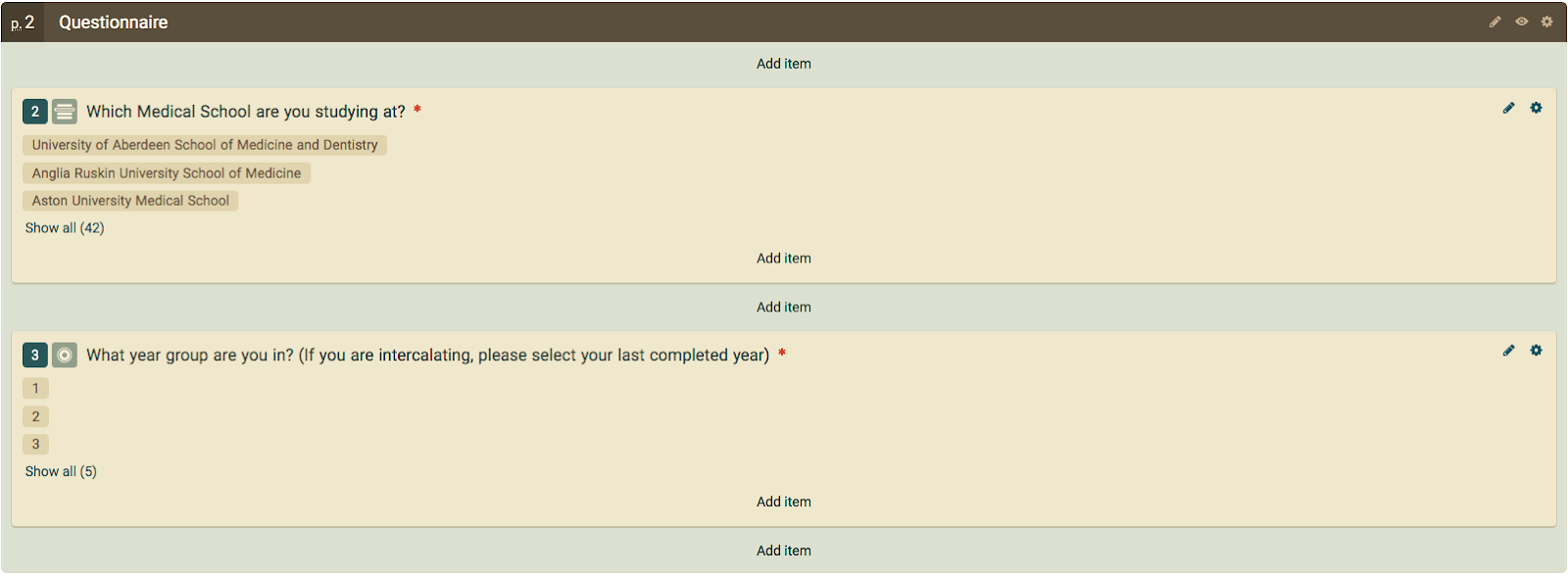
**

**
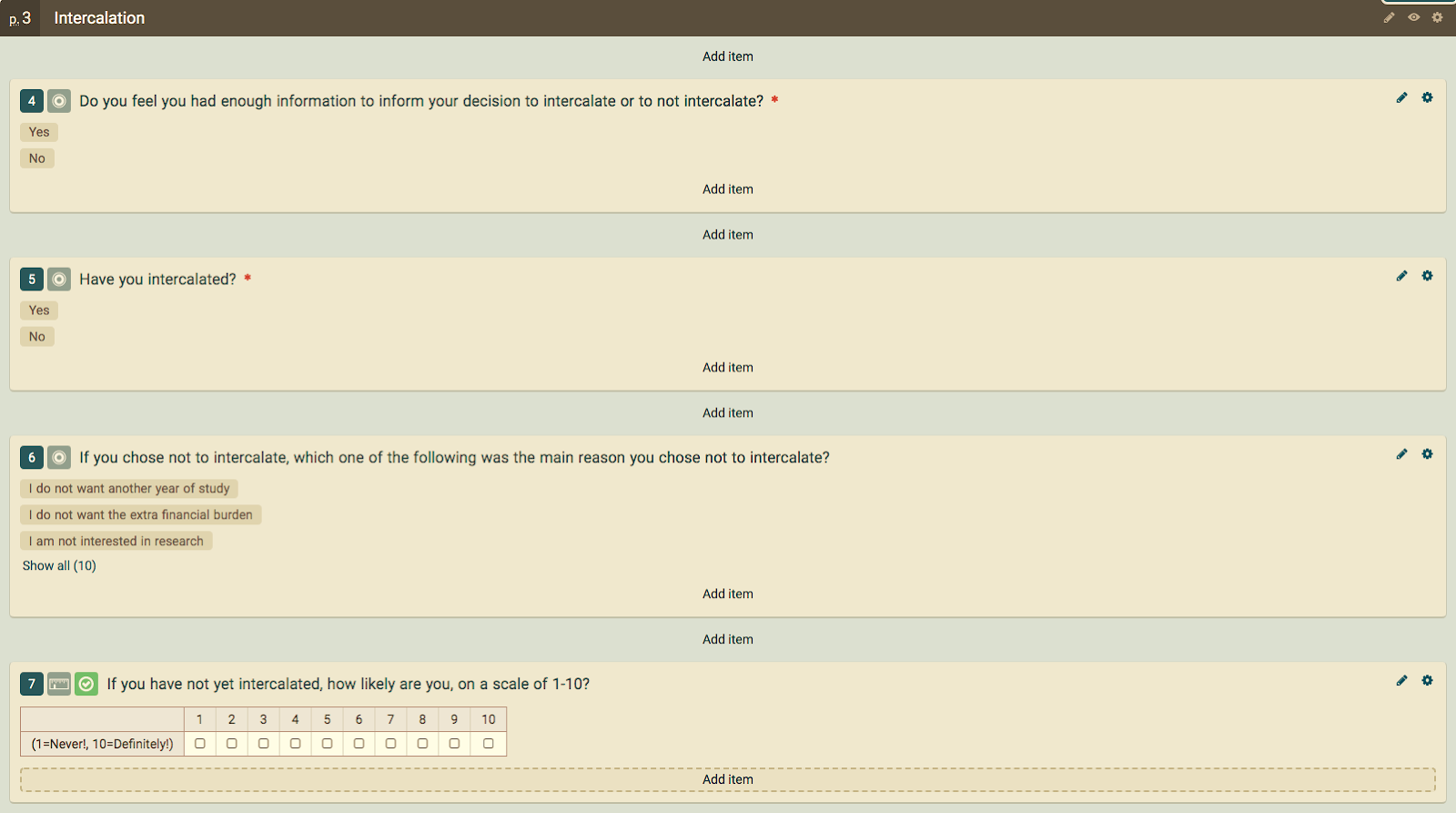
**

**
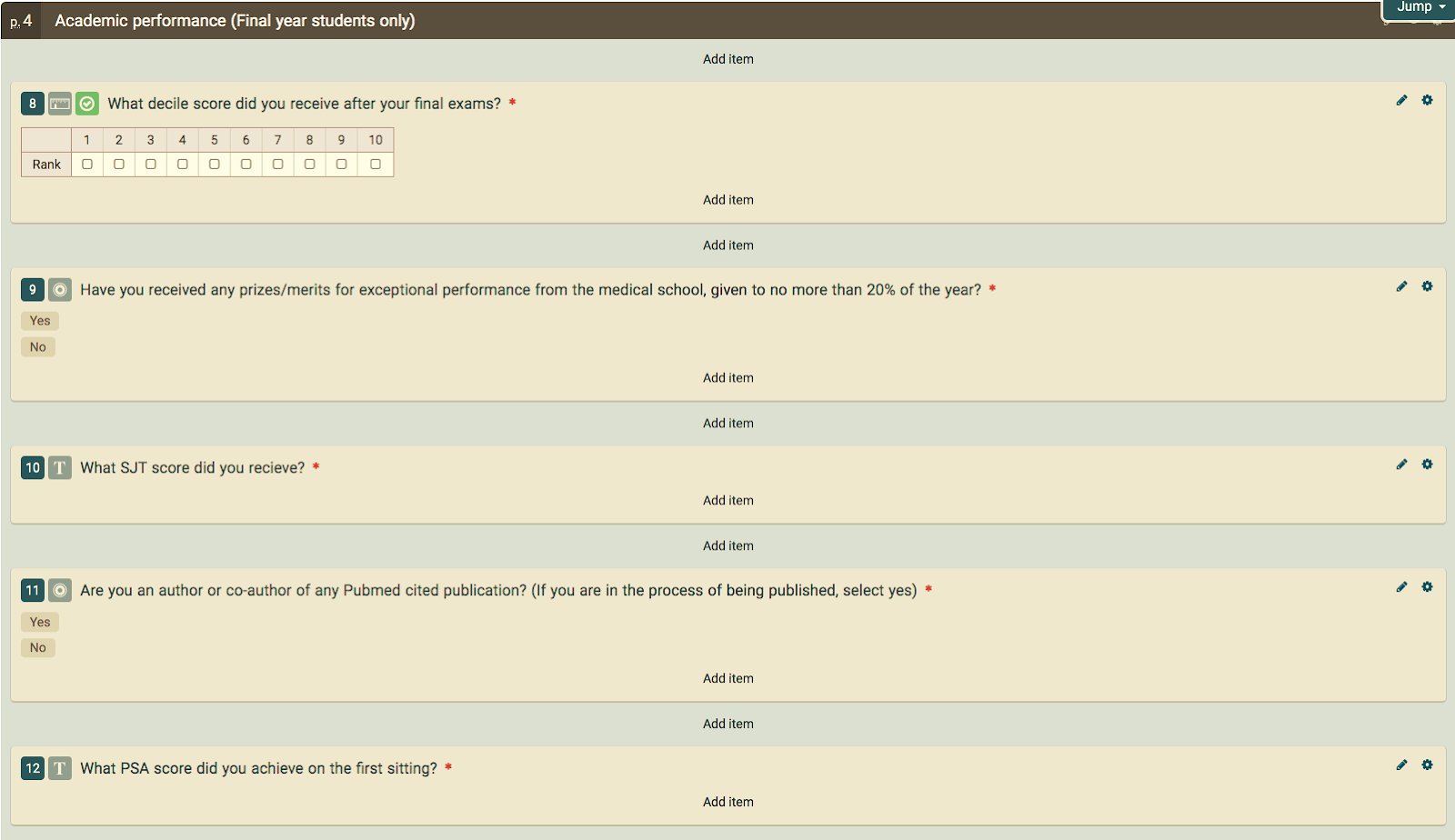
**

**
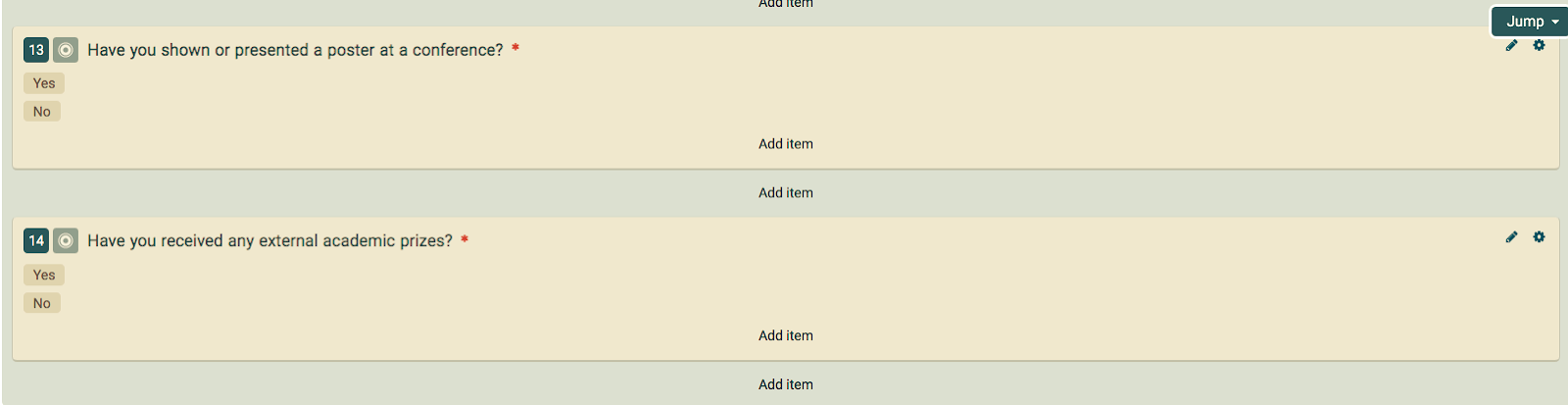
**

**
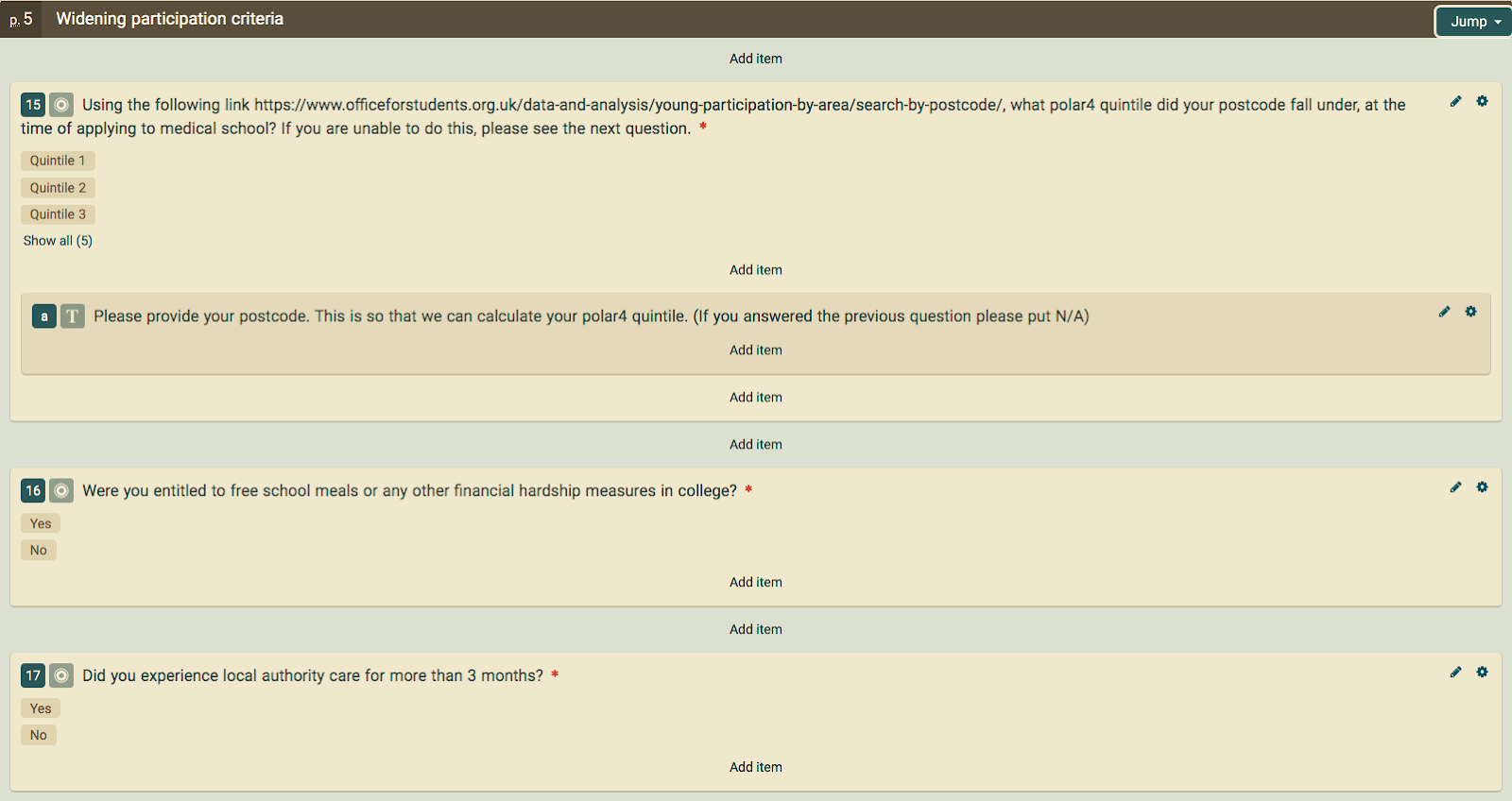
**

**
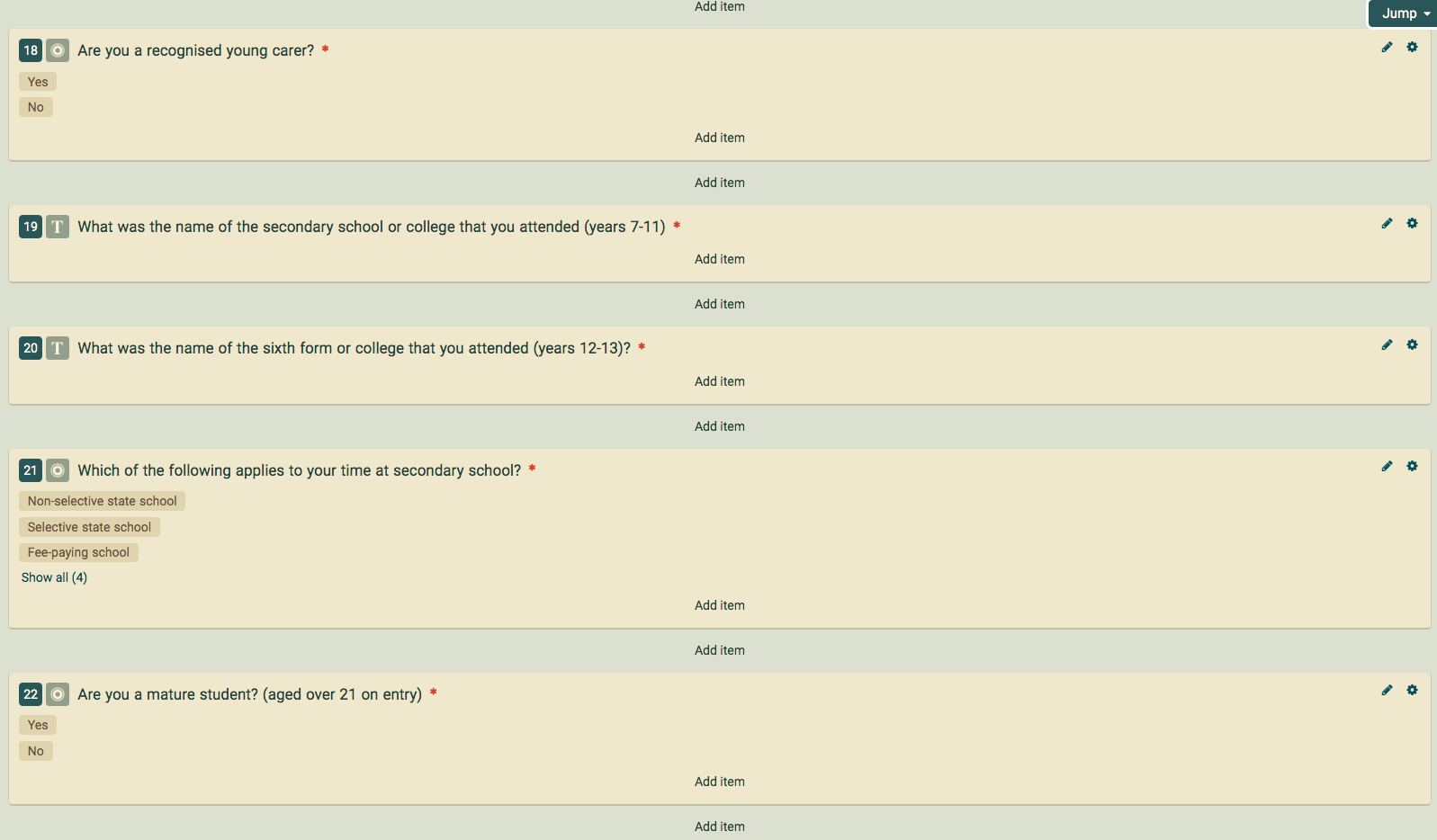
**

**
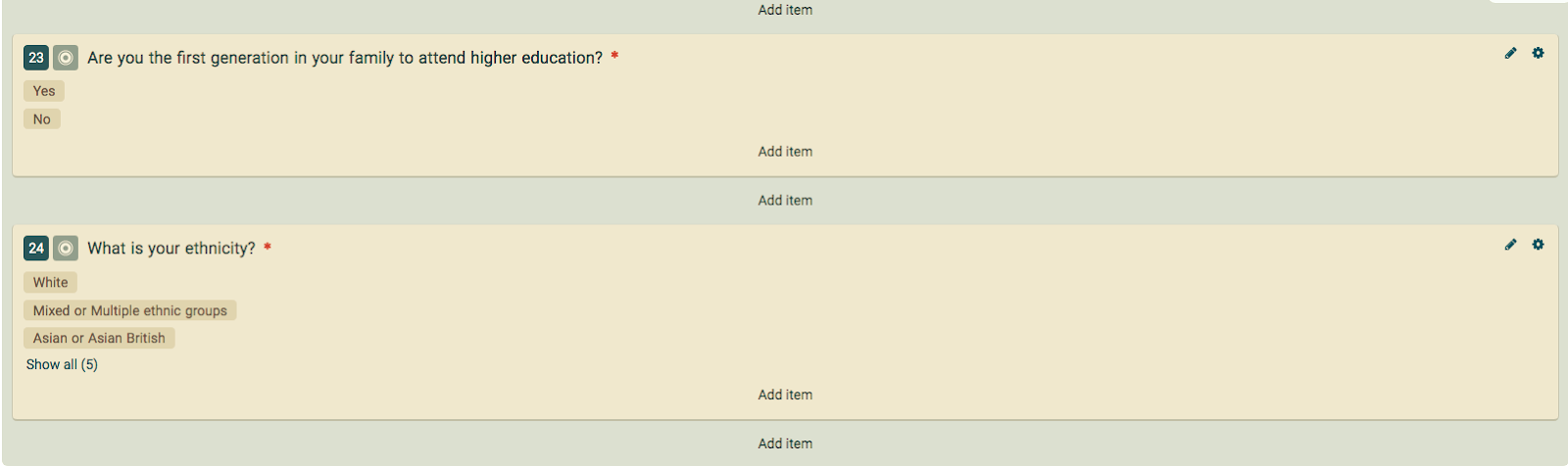
**

**
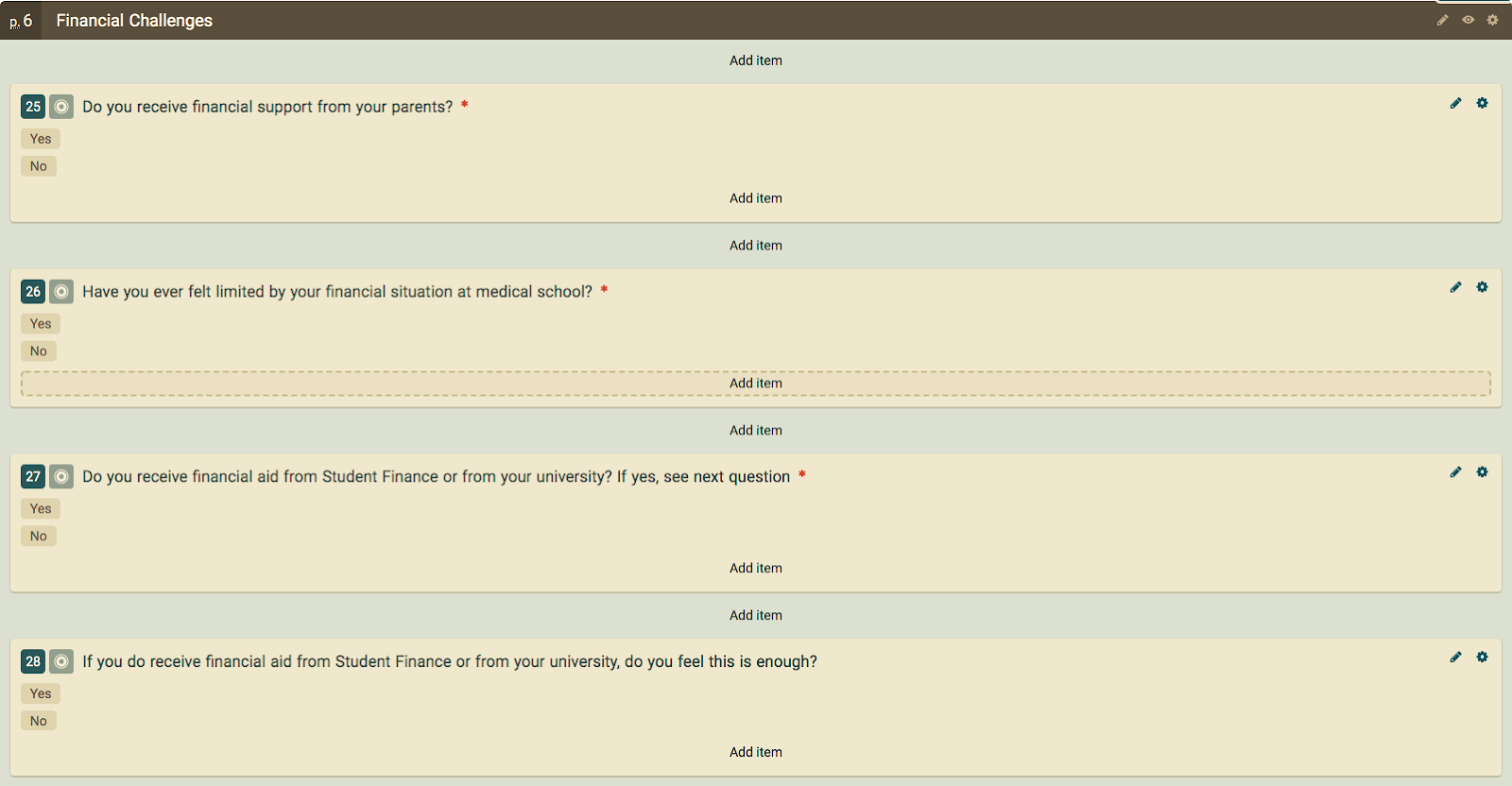
**

**
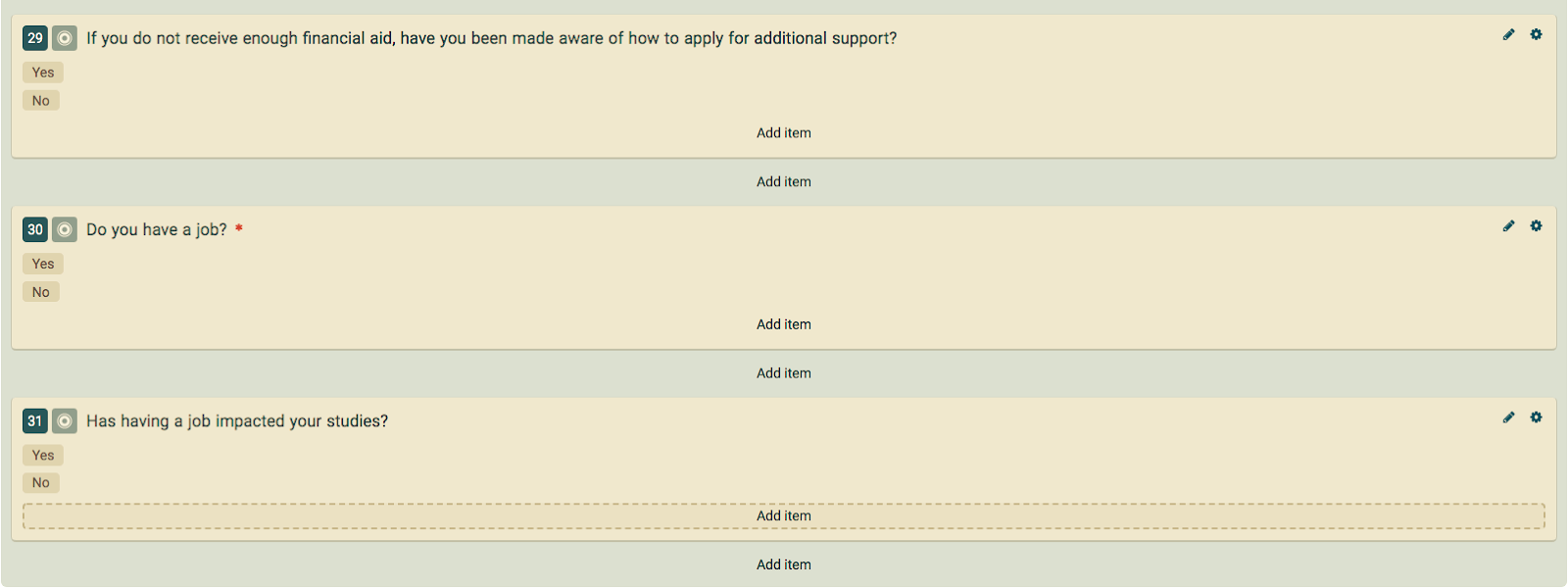
**

**
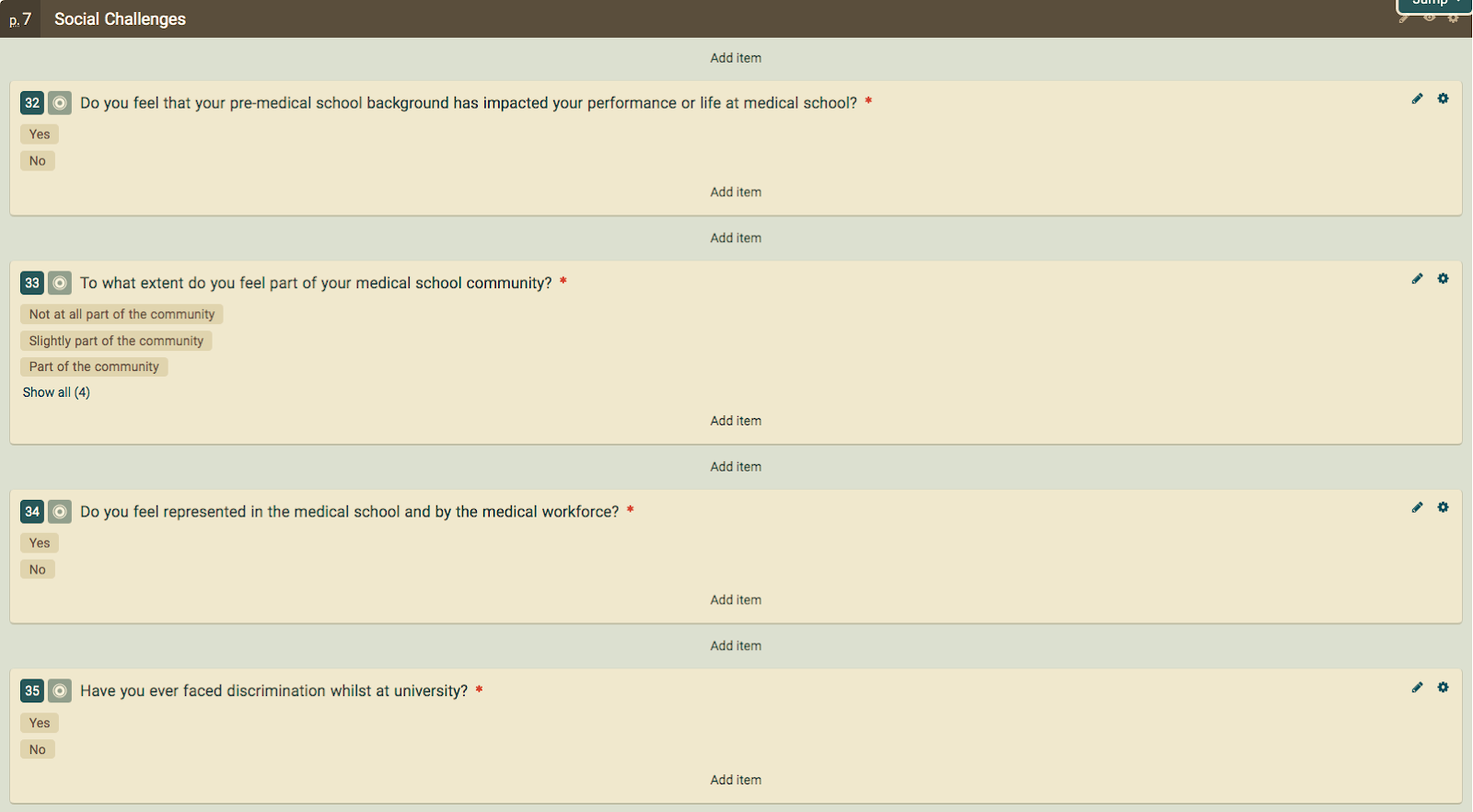
**

**
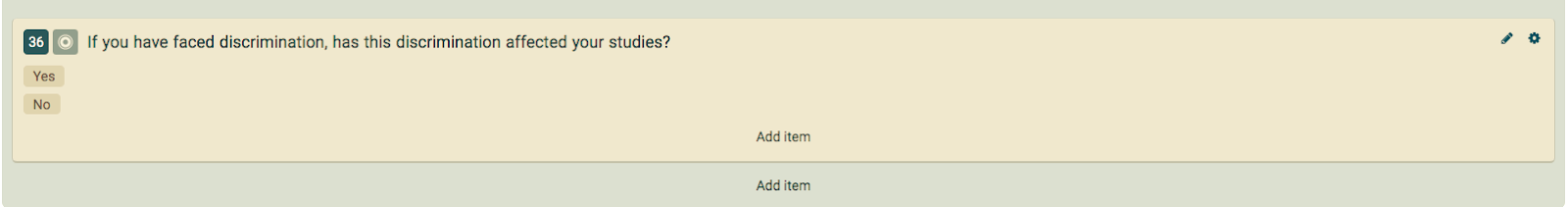
**

**
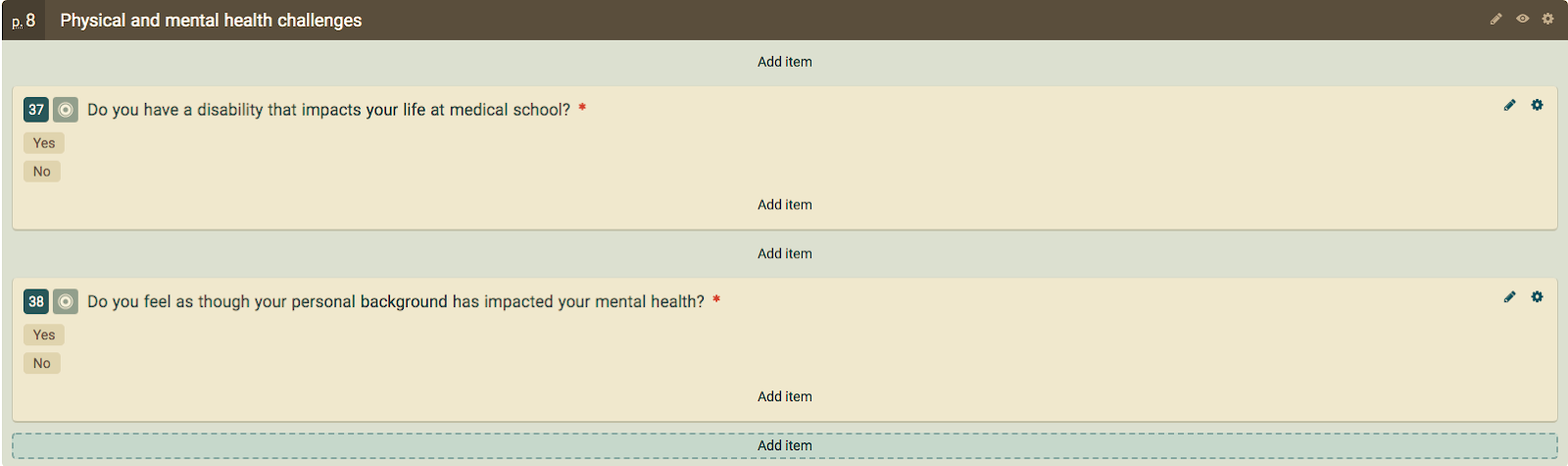
**

**
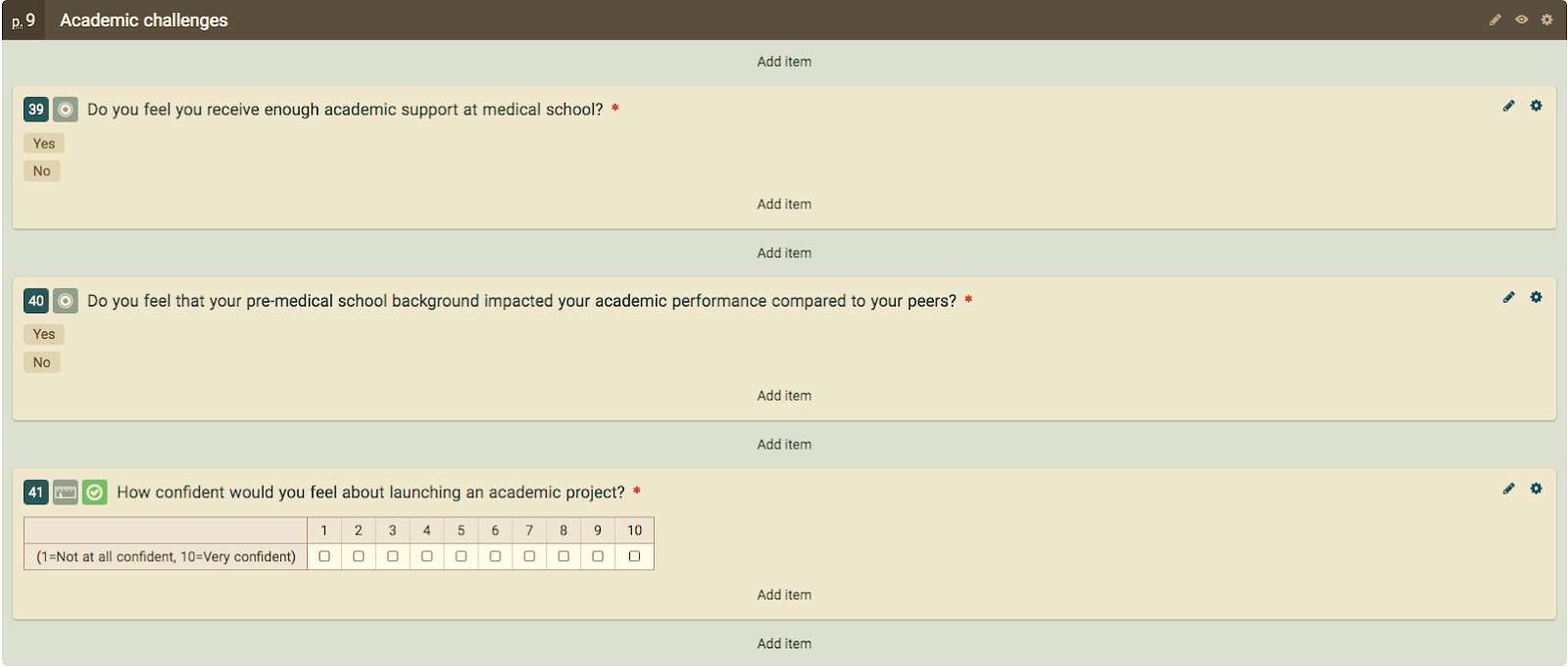
**

**
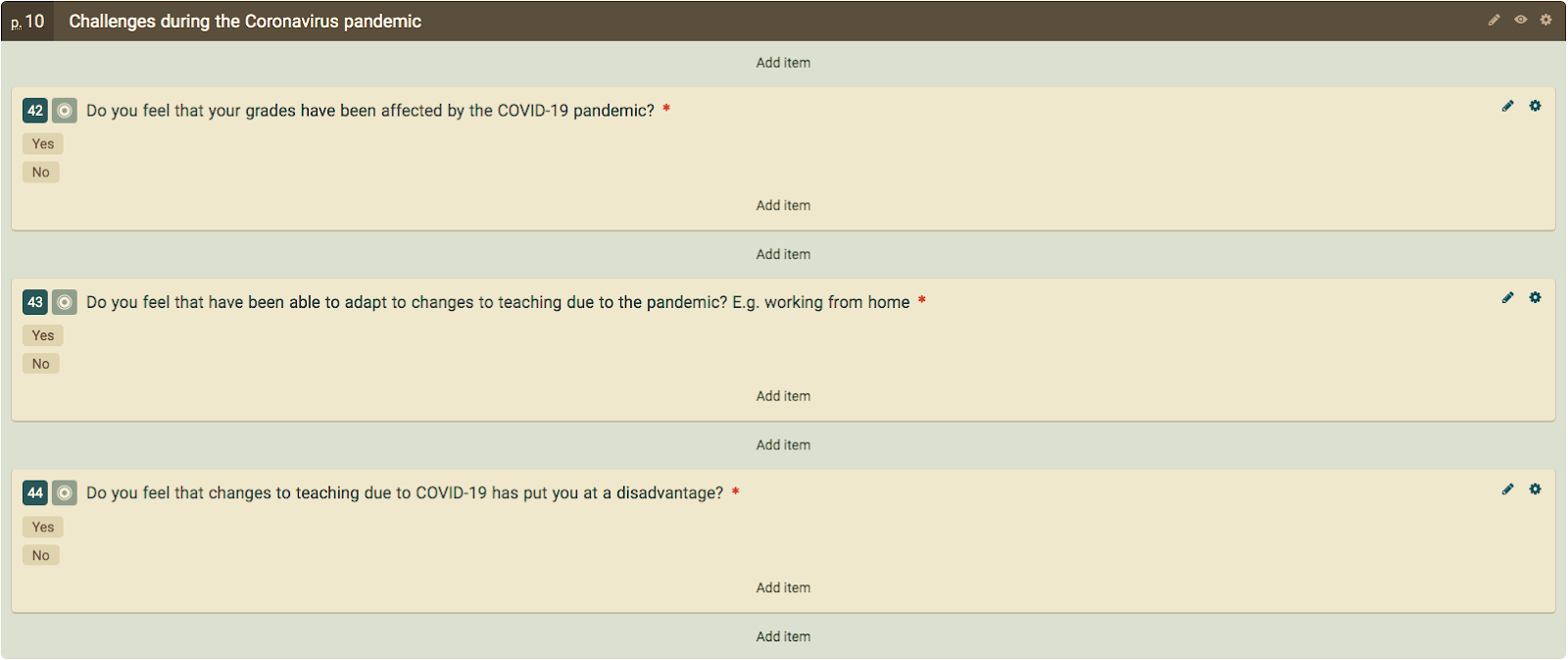
**


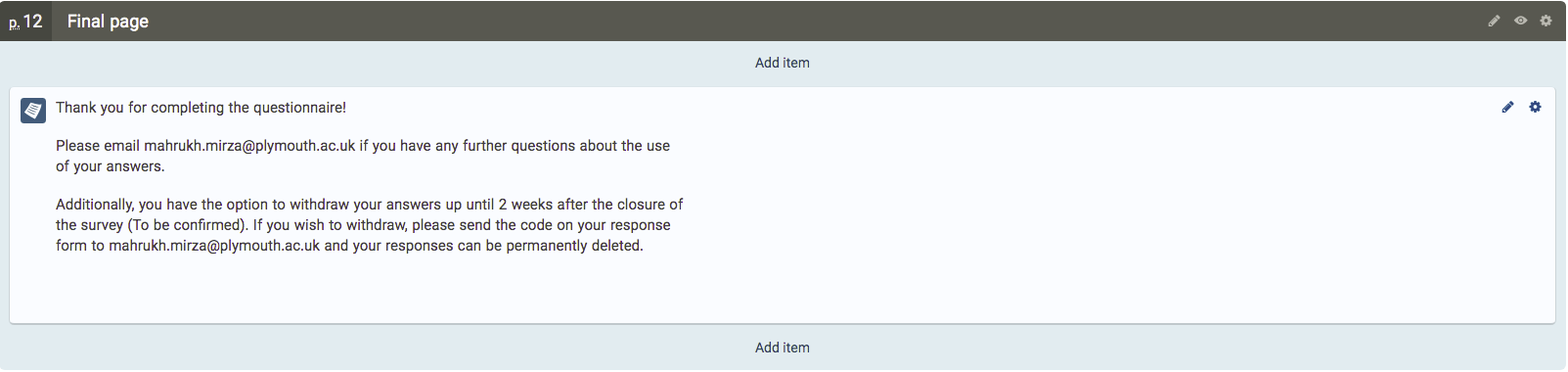

Supplement: sj-docx-1-mde-10.1177_23821205241249012 - Supplemental material for Quantitative Analysis of Challenges Encountered by UK Widening Participation Medical Students in Comparison With Their Non-Widening Participation Peers [file sj-docx-1-mde-10.1177_23821205241249012.docx]
